# Induced DNA bending by unique dimerization of HigA antitoxin

**DOI:** 10.1107/S2052252520006466

**Published:** 2020-06-26

**Authors:** Jin-Young Park, Hyo Jung Kim, Chinar Pathak, Hye-Jin Yoon, Do-Hee Kim, Sung Jean Park, Bong-Jin Lee

**Affiliations:** a Research Institute of Pharmaceutical Sciences, College of Pharmacy, Seoul National University, Seoul 08826, Republic of Korea; b College of Pharmacy, Woosuk University, Wanju 55338, Republic of Korea; c Leicester Institute of Structural and Chemical Biology, University of Leicester, United Kingdom; d Department of Chemistry, Seoul National University, Seoul 08826, Republic of Korea; e College of Pharmacy, Jeju National University, Jeju 63243, Republic of Korea; f College of Pharmacy and Gachon Institute of Pharmaceutical Sciences, Gachon University, 534-2 Yeonsu-dong,Yeonsu-gu, Incheon 13120, Republic of Korea

**Keywords:** *Mycobacterium tuberculosis*, TA system, antitoxins, HigBA, DNA, X-ray crystallography, NMR spectroscopy, structure determination, protein structures

## Abstract

The bacterial toxin–antitoxin (TA) system regulates cell growth under various environmental stresses. *Mycobacterium tuberculosis*, the causative pathogen of tuberculosis (TB), has three HigBA type II TA systems with reverse gene organization, consisting of the toxin protein HigB and labile antitoxin protein HigA. Most type II TA modules are transcriptionally autoregulated by the antitoxin itself. In this report, we first present the crystal structure of the *M. tuberculosis* HigA3 antitoxin (*Mt*HigA3) and *Mt*HigA3 bound to its operator DNA complex. We also investigated the interaction between *Mt*HigA3 and DNA using NMR spectroscopy. The *Mt*HigA3 antitoxin structure is a homodimer that contains a structurally well conserved DNA-binding domain at the N-terminus and a dimerization domain at the C-terminus. Upon comparing the HigA homologue structures, a distinct difference was found in the C-terminal region that possesses the β-lid, and diverse orientations of two helix–turn–helix (HTH) motifs from HigA homologue dimers were observed. The structure of *Mt*HigA3 bound to DNA reveals that the promoter DNA is bound to two HTH motifs of the *Mt*HigA3 dimer presenting 46.5° bending, and the distance between the two HTH motifs of each *Mt*HigA3 monomer was increased in *Mt*HigA3 bound to DNA. The β-lid, which is found only in the tertiary structure of *Mt*HigA3 among the HigA homologues, causes the formation of a tight dimerization network and leads to a unique arrangement for dimer formation that is related to the curvature of the bound DNA. This work could contribute to the understanding of the HigBA system of *M. tuberculosis* at the atomic level and may contribute to the development of new antibiotics for TB treatment.

## Abstract 2

The crystal structures of HigA3 antitoxin and DNA-bound HigA3 allow detailed structural insights into the HigBA toxin–antitoxin system of *Mycobacterium tuberculosis*. The unique dimerization of HigA3 induces DNA bending.

## 1. Introduction   

Tuberculosis (TB) is one of the major infectious diseases caused by the human pathogenic bacterium *Mycobacterium tuberculosis*, affecting 23% of the global population (Lönnroth & Raviglione, 2008 ▸). It is one of the top ten risk factors for death and the leading cause of death from a single infectious agent (above HIV/AIDS). The WHO estimated that 10 million people were infected with TB in 2017, among which 5–15% of this population developed active TB, resulting in a mortality rate of 1.3 million people worldwide in a single year (WHO 2017 data; WHO, 2018 ▸). However, the current regimens of antibiotic combinations have led to resistance, and concerns over tolerance are increasing rapidly. The emergence of multidrug-resistant TB (MDR-TB), which is the cause of TB treatment failure, has been a public health crisis for the last decade. In 2017, approximately 450 000 people developed MDR-TB, and the treatment success rate for MDR-TB was only 55% globally (WHO, 2018 ▸). During the treatment, TB enters a dormancy state that is linked to the antibiotic tolerance of TB due to the capability of persister cell formation from active cells (Torrey *et al.*, 2016 ▸). These persister cells make up a small fraction of the total cells in the initial infection, but become a significant fraction during the dormancy state (Torrey *et al.*, 2016 ▸; Wood *et al.*, 2013 ▸). However, studies on the persister cell formation for the effective treatment of TB have been poorly performed. It is well known that toxin–antitoxin (TA) systems play an important role in both antibiotic resistance via multi-resistant plasmid maintenance and antibiotic tolerance via persister cell formation (Page & Peti, 2016 ▸). In addition, it has been reported that *M. tuberculosis* has approximately 88 TA modules, while non-pathogenic bacteria such as *Mycobacterium smegmatis* have only 4 TA modules (Sala *et al.*, 2014 ▸; Ramage *et al.*, 2009 ▸). Therefore, the importance of TA modules in TB pathogenicity should be emphasized.

TA modules are autoregulated operons that are abundant in the plasmids and chromosomes of many pathogenic bacteria. TA systems regulate the growth of cells responding to a wide variety of stressful environments such as nutrient starvation, antibiotic exposure, heat shock and DNA damage (Pandey & Gerdes, 2005 ▸; Yamaguchi *et al.*, 2011 ▸). TA operons consist of two linked genes in which one encodes a stable toxin protein that inhibits cell growth and the other encodes a cognate antitoxin, such as an antisense RNA or protein that neutralizes the toxin activity (Greenfield *et al.*, 2000 ▸). Toxins show enzymatic functions that interfere with various vital processes of bacteria. Toxins have diverse targets, such as DNA replication, mRNA stability, protein translation, cell wall biosynthesis and ATP synthesis (Yamaguchi *et al.*, 2011 ▸). Activation of toxins is prevented by the binding of antitoxin under normal conditions. However, under stressful conditions, antitoxins are degraded by proteases, and the free toxin is activated (Brzozowska & Zielenkiewicz, 2013 ▸). Broadly, TA systems are classified into six types depending upon the type of antitoxin and how the antitoxin inhibits toxin activity (Page & Peti, 2016 ▸). Among these systems, the type II TA system is one of the well established TA systems; it encodes a stable toxin protein and a labile antitoxin protein, which form a stable TA complex to inhibit the function of the toxin in normal cells (Yamaguchi & Inouye, 2009 ▸). Antitoxins are usually flexible and unstructured in free form but are continuously produced to neutralize the toxicity of toxins (Aizenman *et al.*, 1996 ▸). In most cases, antitoxins bind to the promoters located upstream of the corresponding TA operon to repress transcription. In addition, the TA complex itself acts as a repressor of TA operon transcription by binding to the promoter region, resulting in autoregulation of the TA module (Marianovsky *et al.*, 2001 ▸; Kedzierska *et al.*, 2007 ▸). Post-segregational killing (PSK), abortive infection and bacterial persistence have been reported as the major biological functions of TA modules (Harms *et al.*, 2018 ▸). Among these functions, bacterial persistence is strongly associated with the type II TA system (Page & Peti, 2016 ▸). Upon antitoxin degradation, the free toxin induces the formation of persister cells, leading to the dormancy state of cells. The unusually wide distribution of the type II TA system in *M. tuberculosis* and dormancy of TB may be related. Therefore, the TA system could be a candidate for a new antibiotics target for TB treatment.

The representative type II TA families of *M. tuberculosis* are VapBC, MazEF, ParDE, RelBE and HigBA. To date, diverse structural studies of the type II TA system have been reported, but the structural studies of HigBA in *M. tuberculosis* are limited. The first *higBA* module discovered was on the Rts1 plasmid from *Proteus vulgaris* (Tian *et al.*, 1996 ▸). The *higBA* module differs from other type II TA systems in that the toxin gene *higB* is located upstream of the antitoxin gene *higA.* There are three *higBA* loci (*higBA*1–3) in *M. tuberculosis*, and these loci are highly expressed under multiple stress conditions, such as drug-induced and environmental stress (Gupta *et al.*, 2017 ▸). The HigB toxin is a ribosome-dependent ribonuclease that cleaves adenosine-rich mRNA (Hurley & Woychik, 2009 ▸). This ribonuclease activity inhibits protein transcription following cell growth arrest, which has been reported for several bacteria, including *M. tuberculosis* (Schuessler *et al.*, 2013 ▸). The HigA antitoxin of *M. tuberculosis* (*Mt*HigA) has a Cro/C1-type helix–turn–helix (HTH) domain, which is a common DNA-binding domain existing in prokary­otes and eukaryotes (Luscombe *et al.*, 2000 ▸). This domain is named after the transcription repressors Cro and C1 of bacteriophages 434 and lambda (Aggarwal *et al.*, 1988 ▸). The Cro/C1-type HTH domain-containing proteins function mainly as homodimers and consist of two domains: a bundle of five N-terminal helices that serves a stabilizing hydrophobic core and a C-terminal domain mediating dimerization (Luscombe *et al.*, 2000 ▸). The α2- and α3-helices of the N-terminal helical bundle compose the HTH motif, which is typically defined as a 20-amino-acid segment with two perpendicular α-helices. The α3-helix recognizes the DNA helix.

In this study, we determined the crystal structure of the *Mt*HigA3 antitoxin alone, and the *Mt*HigA3 bound to the operator DNA. Compared with previously known structures of HigA from other species, *Mt*HigA3 shows unique characteristics, including a dimerization interface. Upon forming a complex with promoter DNA, the quaternary structure of *Mt*HigA3 and the curvature of DNA bending seem to be closely related with each other. The interaction between *Mt*HigA3 and the operator DNA in the solution state was investigated by NMR titration experiments which provided additional information about the binding mechanism between DNA and *Mt*HigA3. Upon binding to DNA, residues responsible for dimerization showed major changes along with the HTH motif. Based on this study, we could conclude that the unique dimerization mode of *Mt*HigA3 determines the arrangement of HTH motifs. The position of HTH motifs for each monomer facilitates the sensing of the promoter DNA, resulting in DNA bending. This work could contribute to the understanding of the HigBA system of *M. tuberculosis* on the atomic level, and moreover, may contribute to the development of new antibiotics for TB treatment.

## 2. Materials and methods   

### 2.1. Gene cloning, protein purification and mass spectrometry   

The genes encoding *M. tuberculosis* HigA3 antitoxin (*Mt*HigA3) and 34 residues truncated from the N-terminus of *Mt*HigA3 (*Mt*HigA3^35–117^) were amplified from the *M. tuberculosis* strain H37*Rv* genomic DNA by polymerase chain reaction (PCR). The restriction enzymes used for PCR were Nde1 and Xho1, and the oligonucleotide primers used in this study are listed in Table S1 of the supporting information. The amplified DNA of *Mt*HigA3 and *Mt*HigA3^35–117^ digested by NdeI and XhoI were ligated to the predigested expression vector pET-21*a*(+) (Novagen) containing a C-terminal hexa-histidine tag (LEHHHHHH). Each recombinant plasmid was transformed into *Escherichia coli* DH5α competent cells. The resulting recombinant plasmids were verified by DNA sequencing.

*Mt*HigA3 and *Mt*HigA3^35–117^ cloned into recombinant plasmids were transformed into *E. coli* Rosetta (DE3) and *E. coli* BL21 (DE3) competent cells, respectively. The cells were allowed to grow in Luria broth (LB) medium at 37°C with 50 µg ml^−1^ ampicillin until the OD_600_ reached 0.5. Then, protein expression was induced by the addition of 0.5 m*M* isopropyl β-d-1-thiogalactopyranoside (IPTG). The cells were grown at 37°C for an additional 4 h after induction and were harvested by centrifugation at 5600*g* and 4°C. The cells were resuspended and lysed on ice by sonication in lysis buffer A [50 m*M* Tris–HCl, pH 7.5 and 500 m*M* NaCl containing 10%(*v*/*v*) glycerol]. The cell lysate was centrifuged at 20 000*g* for 1 h at 4°C. The supernatant was applied to an affinity chromatography nickel–nitrilotriacetic acid (Ni-NTA) column (Novagen) previously equilibrated with buffer A. The column was washed with buffer A containing 40 m*M* imidazole, and the protein of interest was eluted with buffer A containing 300 m*M* imidazole. The protein was further purified by size-exclusion chromatography (SEC) using a Superdex 75 (10/300GL) column (GE Healthcare) that was previously equilibrated with 20 m*M* citric acid, pH 5.0 and 150 m*M* NaCl containing 2 m*M* β-mercaptoethanol. The purity of the recombinant *Mt*HigA3 protein was verified by SDS–PAGE, and the protein was concentrated to 10 mg ml^−1^ using an Amicon Ultra centrifugal filter unit (Millipore) for crystallization.

For co-crystallization of *Mt*HigA3 bound to DNA, the protein was incubated with a 20 bp double-stranded DNA (dsDNA; 5′-CCACGAGATATAACCTAGAG-3′) in a 1:4 molar ratio (protein:dsDNA) for 1 h at 20°C before being subjected to size-exclusion column chromatography. For selenomethionine (SeMet) labelling of *Mt*HigA3, the cells were allowed to grow in M9 minimal medium supplemented with SeMet (50 mg l^−1^). The *E. coli* cells containing the recombinant plasmid were grown overnight at 37°C in 10 ml of M9 medium that contained uniformly labelled ^15^N ammonium chloride [U-^15^N] (Cambridge Isotope Laboratory) as a nitrogen source and/or ^13^C glucose [U-^13^C] (Cambridge Isotope Laboratory) as a carbon source. This culture was then inoculated into 1 l of fresh M9 medium supplemented with [U-^15^N] for ^15^N-labelled *Mt*HigA3^35–117^ or with [U-^15^N] and [U-^13^C] for ^15^N,^13^C-labelled *Mt*HigA3^35–117^. The process for protein isolation and purification was the same as described above for non-labelled protein. The final buffer for all NMR samples contained 20 m*M* citric acid, pH 5.0, 150 m*M* NaCl and 1 m*M* DTT. 10%(*v*/*v*) D_2_O was added to the protein samples for NMR to provide the NMR internal lock signal.

*Mt*HigA3 and the matrix solution (5 mg ml^−1^ α-cyano-4-hydroxycinnamic acid in 50% acetonitrile/0.1% TFA) were loaded onto a Teflon-masked MALDI-TOF target. MALDI-TOF MS was performed on a Voyager DETM Biospectrometry Workstation (Applied Biosystems) with a 337 nm nitrogen laser and operating in positive mode at the National Center for Inter-University Research Facilities, Seoul National University (NCIRF).

### 2.2. Crystallization, X-ray data collection and structure determination   

Crystals of *Mt*HigA3, SeMet-labelled *Mt*HigA3 and *Mt*HigA3 bound to DNA were grown by the sitting-drop vapour diffusion method at 293 K using 96-well crystallization plates. Initial crystallization conditions were established using screening kits from Hampton Research (Crystal Screens I and II, Index, PEG/Ion and Natrix), Molecular Dimensions (Memgold I and II, Structure Screen I and II and Proplex) and Emerald BioSystems (Wizard I, II, III and IV) by mixing 0.5 µl of protein solution with 0.5 µl of reservoir solution. The best crystals of *Mt*HigA3 and SeMet-labelled *Mt*HigA3 were obtained from a reservoir solution containing 1 *M* ammonium sulfate, 0.1 *M* HEPES and 0.5%(*w*/*v*) PEG8000. The best crystals of *Mt*HigA3 bound to the DNA complex were obtained from a reservoir solution containing 23%(*w*/*v*) PEG3350 and 0.2 *M* sodium acetate trihydrate pH 7.0. For crystal freezing, the crystals were transferred to a cryoprotectant solution with 20%(*v*/*v*) glycerol under crystallization conditions prior to freezing and mounting. The X-ray diffraction data for the crystals of *Mt*HigA3 and *Mt*HigA3 bound to DNA were collected at 100 K using an ADSC Quantum 315r CCD detector system (Area Detector Systems Corporation) at the BL-5C experimental station of Pohang Light Source, Korea. X-ray diffraction data for the crystals of SeMet-labelled *Mt*HigA3 were collected at 100 K using a PILATUS 6M detector system (Dectris) at the BL-11C experimental station of Pohang Light Source, Korea. For each image, the crystal was rotated by 1° and the raw data were processed and scaled using the program suite *HKL2000* (Otwinowski & Minor, 1997 ▸). Further data analysis was carried out using the *CCP*4 suite and *Phenix* (Winn *et al.*, 2011 ▸; Adams *et al.*, 2010 ▸).

To determine the *Mt*HigA3 structure, the SAD dataset was used and the *Autosol* and *Autobuild* Wizards in the *Phenix* package were employed (Adams *et al.*, 2010 ▸). The four selenium sites were identified and the initial phases calculated from these sites were further improved by density modification. The structure of *Mt*HigA3 bound to DNA was determined using molecular replacement with the program *PhaserMR* within the *Phenix* package (Adams *et al.*, 2010 ▸; Vagin & Teplyakov, 2010 ▸). Iterative cycles of model building were performed using *Coot* (Emsley *et al.*, 2010 ▸), followed by refinement with *REFMAC5* (Murshudov *et al.*, 2011 ▸). The *Mt*HigA3 crystal structure belongs to space group *I*4 and contains two molecules per asymmetric unit. The crystal structure of *Mt*HigA3 bound to DNA was determined to be *C*2 with dimeric *Mt*HigA3 and pseudo-palindromic DNA. A portion of the data (5%) was set aside for the refinement calculations of *R*_free_. The data collection and final crystallographic statistics are summarized in Table S2. The protein interface area was calculated using the *PISA* server (Krissinel & Henrick, 2007 ▸), and sequence alignments were performed using *ClustalX* (Larkin *et al.*, 2007 ▸) and visualized by *ESPript* (Robert & Gouet, 2014 ▸). The electrostatic surface was calculated using the program *APBS* (Baker *et al.*, 2001 ▸), and all figures were generated using *PyMol* version 1.8 (Schrodinger LLC) and *Chimera* (Pettersen *et al.*, 2004 ▸).

### 2.3. Electrophoretic mobility shift assay   

Electrophoretic mobility shift assay (EMSA) was performed to characterize the binding of *Mt*HigA3 to the promoter region of the DNA. To search for potential DNA sequences that may be important for TA systems, bioinformatics tools were used. The putative promoter regions for *Mt*HigBA3 and *Mt*HigA3 were searched for using the *BPROM* tool (Solovyev, 2011 ▸), and palindromic DNA sequences were searched for using the *EMBOSS* palindrome tool (http://www.bioinformatics.nl/cgi-bin/emboss/palindrome). The annealed dsDNA for two putative operator sites (DNA-1, 5′-CCGACGATGACCGCGCAC-3′ and DNA-2, 5′-CCA­CGAGATATAACCTAGAG-3′) and two palindromic sequences in the promoter region (DNA-3, 5′-GCCGAG­CAGGCTGCCTCGTTCCTGCTCGGT-3′ and DNA-4, 5′-AGATATAACCTAGAGGTTATACTG-3′) were purchased from Bioneer Innovation (http://www.bioneer.co.kr). The *Mt*HigA3 protein and dsDNAs were prepared in buffer consisting of 20 m*M* citric acid, pH 5.0 and 150 m*M* NaCl. The dsDNA (0.01 µ*M*) was allowed to equilibrate with increasing concentrations of protein (apo, 0.1, 0.2, 0.3, 0.4, 0.5 and 0.6 µ*M*) for 30 min at 20°C before loading on a 0.8% agarose gel in 0.5 TBE (45 m*M* Tris–borate and 1 m*M* EDTA) buffer. The results were visualized using a Gel Doc (Bio-Rad).

### 2.4. Isothermal titration calorimetry measurements   

Isothermal titration calorimetry (ITC) experiments were performed using a MicroCal 200 (GE Healthcare) at 25°C, and all buffers, reagents and proteins were degassed prior to use. *Mt*HigA3 and dsDNAs were prepared in a buffer consisting of 50 m*M* sodium acetate, pH 5.2 and 150 m*M* NaCl. *Mt*HigA3 (50 µ*M*) was titrated into the experimental chamber containing 3.3 µ*M* DNA. Measurements were conducted with an injection volume of 0.4 µl as an initial injection volume followed by 2 µl for the remaining 19 injections with 150 s spacing and a stirring speed of 750 rev min^−1^ at 25°C. The heat signals obtained from the raw ITC data were analysed with the *Origin* software, supplied by MicroCal Inc., fitting the data into a single-site binding isotherm to obtain the binding affinity (*K*_d_), the change in enthalpy (Δ*H*) and the change in entropy (Δ*S*). The Gibbs free energy (Δ*G*) was calculated using the standard equation Δ*G* = Δ*H* − *T*Δ*S.*

### 2.5. NMR spectroscopy and chemical shift perturbation experiments   

All NMR spectra of ^15^N-labelled *Mt*HigA3^35–117^ and ^15^N,^13^C-labelled *Mt*HigA3^35–117^ were obtained on a Bruker Avance 800 MHz spectrometer equipped with a cryoprobe. All NMR samples were dissolved in the final buffer (20 m*M* citric acid, pH 5.0 and 150 m*M* NaCl containing 1 m*M* DTT), and all experiments were performed at 298 K. Data for the carbonyl carbon were obtained using the HNCO and HNCACO spectra, and data for the α/β carbon were acquired using the HNCA, HNCOCA, HNCACB and CBCACONH spectra. The δ p.p.m. values of the backbone N and HN resonances of *Mt*HigA3^35–117^ were assigned using these datasets. The chemical shift perturbation experiments were carried out by titrating U-^15^N labelled *Mt*HigA3^35–117^ with promoter DNA. The phase-sensitive 2D-[^1^H-^15^N] TROSY–HSQC spectrum with sensitivity improvement was acquired for U-^15^N labelled *Mt*HigA3^35–117^ alone and with increasing concentrations of dsDNA (protein:dsDNA in 1:0.05, 1:0.1, 1:0.2 and 1:0.4 molar ratios). Upon titration, the disappearance or shifting of the NMR signal was monitored. All spectra were processed and analysed using *NMRPipe*/*NMRDraw* (Delaglio *et al.*, 1995 ▸) and *NMRView* (Johnson & Blevins, 1994 ▸). The average chemical shift perturbation (CSP) values of ^15^N and ^1^H were calculated using the following equation (Schumann *et al.*, 2007 ▸):
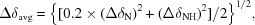
where Δδ_N_ and Δδ_NH_ represent the CSP values of the amide nitrogen and proton, respectively.

## 3. Results   

### 3.1. Crystal structure of *Mt*HigA3   

There are three HigBA systems in *M. tuberculosis*, but structural information is not yet available on the antitoxin HigA. We first determined the crystal structure of the HigA3 antitoxin from *M. tuberculosis* (*Mt*HigA3) to 1.97 Å resolution by single anomalous dispersion using selenomethionine-substituted protein. *Mt*HigA3 was crystallized in the tetragonal space group *I*4 with one *Mt*HigA3 homodimer per asymmetric unit. Although we tried to crystallize *Mt*HigA3 with the full sequence (MALDI–TOF MS confirmed the molecular weight of purified *Mt*HigA3 to be 12.85 kDa, which corresponds to the molecular weight of full-length *Mt*HigA3, see Fig. S1 of the supporting information), the N-terminal residues (1 to 33 or 35) were not defined due to the missing electron density. Hadzi *et al.* previously showed that the N-terminal residues of HigA2 from *Vibrio cholerae* were proteolytically degraded (Hadži *et al.*, 2017 ▸, 2013 ▸). Considering that *Mt*HigA3 is a protease-labile antitoxin and is structurally similar to HigA2 from *V. cholerae*, it is possible that the N-terminus of *Mt*HigA3 is proteolytically degraded during crystallization.

**Figure 1:**
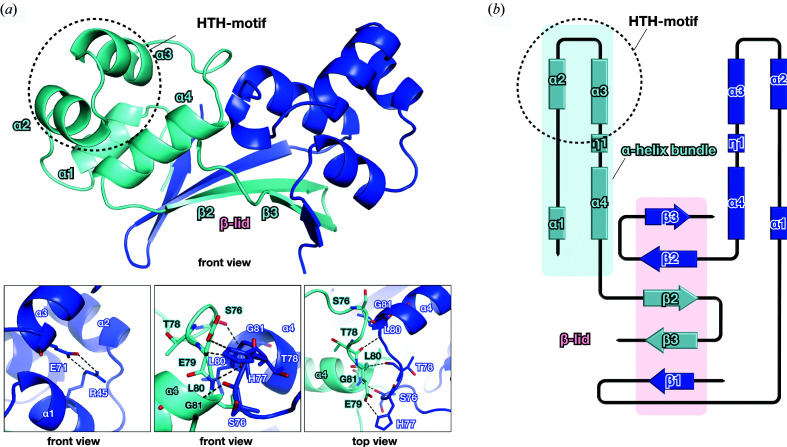
Crystal structure of *Mt*HigA3. (*a*) Front view of the overall *Mt*HigA3 structure. Two monomers of *Mt*HigA3 interact to form a dimer with HTH motifs on either side of the dimer structure, and a β-lid is formed as a result of this dimer organization. Front view of the salt bridge formed between Arg45 and Glu71 (left). Front view (middle) and top view (right) of the hydrophilic interactions between the loop and the adjacent α4-helix are enlarged in the panels. Each monomer, including residues from each chain, is coloured cyan and blue. (*b*) Topology diagram of the *Mt*HigA3 dimer. The secondary structure elements are coloured the same as in (*a*).

The monomer structure of *Mt*HigA3 consists of either two or three β-strands, four α-helices and one 3_10_-helix (η) [Fig. 1 ▸(*a*)]. The topology follows the order β1 (residues 36–38), α1 (residues 41–48), α2 (residues 53–60), α3 (residues 64–72), η1 (residues 75–77), α4 (residues 80–89), β2 (residues 93–100) and β3 (residues 103–106). Four α-helices and one 3_10_-helix together form the α-helix bundle that includes the HTH DNA-binding motif. While forming a dimer, the β1-strand from one monomer is aligned parallel to two antiparallel β-strands (β2 and β3) from the other monomer to form a mixed β-sheet. This intermolecular interaction (mixed β-sheet) forms a ‘β-lid’ on one side of the dimer, while the two HTH motifs responsible for DNA binding flank outwards [Fig. 1 ▸(*b*)].

The SEC results (Fig. S2) show a single peak corresponding to a molecular weight of 26 kDa, suggesting that *Mt*HigA3 forms a dimer in solution. In the crystal structure, each monomer forms a tight dimer with a large interface area (1459.2 Å^2^) accounting for 26% of the total solvent-accessible surface. Both hydrophilic and hydrophobic interactions were included in the dimerization. Hydrophilic interactions can be observed at the loop between η1 and the α4-helix, including residues Ser76, His77, Thr78, Glu79, Leu80 and Gly81, and at the solvent-exposed surface of the β-lid, including residues Val36, Ala38, His93, Arg95, Val97, Glu99, Thr104, Glu106 and Thr108 [Fig. 1 ▸(*a*)]. Notably, the β-lid is highly responsible for the hydrophilic interactions required for dimerization. The hydrophobic interactions involve a dimeric hydrophobic core through Ile44, Ala47 and Leu48 on α1, Leu75 and His77 on η1, Leu83, Val87 and Leu90 on the α4-helix, and the internal residues of the β-lid. This dimerization mode corresponds to typical characteristics of the Cro/C1-type HTH domain: α2- and α3-helices form the HTH motif, and α4- and α5-helices are involved in dimerization, while the α-helix bundle composes a hydrophobic core (Luscombe *et al.*, 2000 ▸). Additionally, residues Arg45 and Glu71 in the α-helix bundle form a salt bridge that improves the stability of the hydrophobic core, including the HTH motif. A well ordered HTH motif is located on the surface of *Mt*HigA3 and exhibits a distinct positive charge on the overall negatively charged surface, facilitating the binding of DNA with *Mt*HigA3.

### 3.2. Structural comparison of *Mt*HigA3 with other homologues   

**Figure 2:**
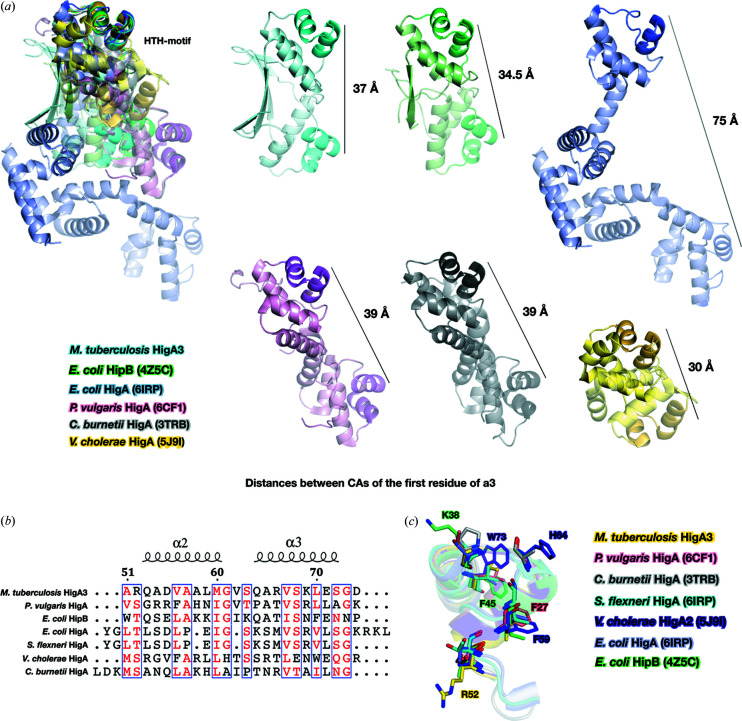
Sequence and structural comparison of *Mt*HigA3 with other homologues. (*a*) Superposition and structural comparison of *Mt*HigA3 with the structures of *E. coli* HipB (lime green, PDB code: 4z5c), *E. coli* HigA (slate, PDB code: 6irp; Yoon *et al.*, 2019 ▸), *P. vulgaris* HigA (pink, PDB code: 6cf1; Schureck *et al.*, 2019 ▸), *C. burnetii* HigA (grey, PDB code: 3trb; Franklin *et al.*, 2015 ▸) and *V. cholerae* HigA (yellow, PDB code: 5j9i; Hadži *et al.*, 2017 ▸). The adjacent chain of each dimer is coloured lighter, and HTH motifs are coloured darker. The distances between CAs of the first residue of the α3-helix are shown as black lines. (*b*) Sequence alignment focusing on the HTH motif region in *M. tuberculosis* HigA3, *P. vulgaris* HigA, *E. coli* HipB, *E. coli* HigA, *S. flexneri* HigA, *V. cholerae* HigA and *C. burnetii* HigA. Similar residues are coloured red in the box. Secondary structural elements of *Mt*HigA3 are presented above the sequence, where the helices are indicated by springs. The residue numbering corresponds to *M. tuberculosis* HigA3. (*c*) Superposition of the HTH motif of *M. tuberculosis* HigA3 (yellow) with the structures of *P. vulgaris* HigA (pink, PDB code: 6cf1), *E. coli* HipB (lime green, PDB code: 4z5c), *E. coli* HigA (slate, PDB code: 6irp), *S. flexneri* HigA (cyan, PDB code: 5ycl), *V. cholerae* HigA (purple blue, PDB code: 5j9i) and *C. burnetii* HigA (grey, PDB code: 3trb). Positively charged residues and aromatic residues are shown as sticks.

To date, structures of HigA have been reported from five species, namely *Coxiella burnetii* (sequence homology 8.6%), *E. coli* (sequence homology 11.5%), *P. vulgaris* (sequence homology 15.4%), *V. cholerae* (sequence homology 16.5%) and *Shigella flexneri* (sequence homology 11.5%). Most HigA structures have been solved as HigBA complexes. In addition, solitary antitoxin structures for *C. burnetii, V. cholerae, P. vulgaris* and *E. coli* are available. The low sequence homology of *Mt*HigA3 with other HigA structures indicates a major difference in its three-dimensional structure. Clearly, all the HigA structures were significantly different except for the HTH motif [Fig. 2 ▸(*a*)]. All the known HigA structures showed only α-helices, while *Mt*HigA3 had an additional two β-strands that participated in dimerization. HigA of *E. coli* (*Ec*HigA) and HigA of *S. flexneri* (*Sf*HigA) show complete conservation of 141 amino acids, composing 9 α-helices. Their entire length is longer than that of *Mt*HigA3, and in contrast to *Mt*HigA3, the HTH motif of *Ec*HigA and *Sf*HigA is located on the C-terminus. The N-terminal α1–α4-helices are responsible for dimerization of *Ec*HigA and *Sf*HigA, and the α3–α6-helices are involved in the formation of complex structures with the corresponding toxins. The structures of HigB toxin-bound HigA from *P. vulgaris* (*Pv*HigA) and from *V. cholerae* (*Vc*HigA2) reveal that the N-termini of both *Pv*HigA and *Vc*HigA2 interact with the corresponding toxins. The structure of *Vc*HigA2 was determined in the HigBA2 complex in addition to the structure of the HigA2 antitoxin alone. Interestingly, 36 residues at the N-terminus in the *Vc*HigA2 antitoxin crystal structure were missing, as observed in *Mt*HigA3, whereas in the *V. cholerae* HigBA2 complex, the whole HigA structure was observed. The N-terminal 36 residues, which were missing in the *Vc*HigA2 solitary structure, contained a single α-helix, a single β-strand and a long loop. This component connects the α-helix bundle and simultaneously interacts with HigB2 of *V. cholerae*.

Since the proteins that have Cro/C1-type HTH domains function mainly as dimers to bind to DNA, their antitoxin quaternary structures are compared. Although HigA antitoxins from different species share the same fold in the α-helix bundle, their dimerization mode varies due to the individual N- and C-terminal secondary structures. Comparison of the distances between the two HTH motifs of each monomer in the HigA dimer structures showed that the distances varied from 30 to 75 Å [Fig. 2 ▸(*a*)]. Additionally, the angles between the central axes of the HigA antitoxin molecules in the dimers varied from 63 to 78° (Fig. S3). The distances of the HTH motifs and the dimer angles determine the orientations of the two HTH motifs from each chain. Therefore, the orientation of the two HTH motifs from each chain is unique, indicating their distinct DNA binding patterns.

To compare the structural similarity, *Mt*HigA3 was analysed using the *DALI* server (Holm & Rosenstrom, 2010 ▸). The *DALI* search revealed the structure of *E. coli* HipB (*Ec*HipB, PDB 4z5h, *DALI* score 7.2, sequence homology 14.6%, r.m.s.d. 2.0) as the highest match to the structure of *Mt*HigA3. High-persistence A (*hipA*) was first discovered in the mutant *E. coli* K-12 in 1983 (Moyed & Bertrand, 1983 ▸). The toxin HipA is an EF-Tu kinase that inhibits protein synthesis, and the antitoxin protein HipB neutralizes toxin activity or is degraded by Lon protease (Correia *et al.*, 2006 ▸; Hansen *et al.*, 2012 ▸). From a structural perspective, the α-helix bundle of *Ec*HipB is more similar to that of *Mt*HigA3 than those of other HigA homologues. *Ec*HipB and *Mt*HigA3 share similar characteristics in the hydrophobic core [Fig. 2 ▸(*a*)]. The C-terminal β-lid, a unique characteristic of *Mt*HigA3, was only observed in *Ec*HipB, but only one β-strand from each domain forms the β-lid for *Ec*HipB. Instead of an additional β-strand, *Ec*HipB has a C-terminal stretch that consists of 16 residues and is unstructured. This C-terminus is recognized as a degradation signal by Lon protease (Hansen *et al.*, 2012 ▸).

Despite the low sequence homology shared among the members of the HigA family, the HTH motif is well conserved [Fig. 2 ▸(*b*)]. Although the sequences of the HTH motifs are not identical, similarities between residues in HTH motifs can be observed in HigA family proteins. Several proteins of this family share positively charged residues that can contribute to DNA recognition, whereas others possess aromatic residues that can contribute to the conformational stability of the DNA–protein complex structure (Anjana *et al.*, 2012 ▸; Cherstvy, 2009 ▸) [Fig. 2 ▸(*c*)]. In the cases of *Ec*HigA and *Sf*HigA, the α2-helix is shorter than those of other homologues, which makes the HTH motif insecure. Another difference is that the aromatic residues are occasionally found in α-helices [Fig. 2 ▸(*c*)]. The aromatic residues are found in Phe27 in the α2-helix of *Pv*HigA, Phe45 in the α3-helix of *Ec*HigA, Phe59 in the α2-helix of *Vc*HigA2 and Trp73 in the α3-helix of *Vc*HigA2. This sequential variation in the HTH motif aids in the recognition of diverse DNA sequences and the stabilization of the DNA–protein complex structure.

### 3.3. Analysis of the HigBA operon of *M. tuberculosis* and identification of the operator site   

**Figure 3:**
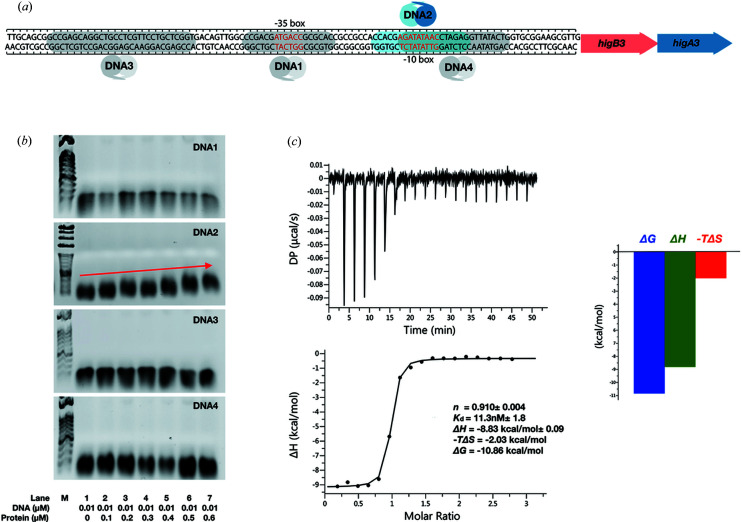
*Mt*HigA3 binds to the *higBA3* promoter region at a specific sequence. (*a*) Organization of the *higBA3* operon. The *higB3* toxin gene is located upstream of the *higA3* antitoxin gene. Two putative operator regions predicted by the *BPROM* web interface (Solovyev, 2011 ▸) located at the −35 and −10 positions are shown in red letters. DNA1, which includes the −35 box, is highlighted as grey, and DNA2, which includes the −10 box, is highlighted in blue. Predicted palindromic sequences (DNA3 and DNA4) for the *higBA3* gene are coloured grey. The locations of the *higB* and *higA* genes are indicated by red and blue arrows, respectively. (*b*) EMSA experiments of the *higBA3* promoter region DNA with increasing concentrations of *Mt*HigA3 (0, 0.1, 0.2, 0.3, 0.5 and 0.6 µ*M*). Only DNA2 is shifted upwards with increasing *Mt*HigA3 concentration. Band shifts are indicated by red narrows. (*c*) ITC measurement upon the interaction of *Mt*HigA3 and DNA2. (Left) Graphical representation of thermodynamic parameters for the *Mt*HigA3 and DNA2 interaction. (Right) *Mt*HigA3 binding to DNA2 is associated with a favourable entropy change.

In most type II TA systems, the antitoxin is located upstream of the toxin. Both the antitoxin and TA complex could bind to the operator DNA of TA modules and autoregulate the TA operon. However, *Mt*HigBA3 has a reverse TA system, and *Mt*HigB3, the cognate toxin of *Mt*HigA3, is located directly upstream of the *Mt*HigA3 antitoxin. We hypothesized that, in this reverse gene order, an additional promoter for the antitoxin could regulate the gene transcription. Therefore, we searched for the putative promoter regions of *Mt*HigBA3 and *Mt*HigA3 using the *BPROM* tool (Solovyev, 2011 ▸). However, unlike the *Acinetobacter baumannii higBA2* operon (Armalyte *et al.*, 2018 ▸), we found only the predicted operator regions of *Mt*HigBA3. In the *A. baumannii higBA2* operon, Armalyte *et al.* found two distinct promoter regions located on the DNA: one for the complete TA system (higBA2) and the other for the *higA2* antitoxin (Armalytė *et al.*, 2018 ▸). The *higBA3* promoter region, 230 bp of the *Mt*HigB3 upstream DNA, includes two putative operator sites: a −35 box (DNA1) and a −10 box (DNA2). We expanded our search to look for any additional DNA interacting regions, and found two palindromic sequences in the promoter region. One of these sequences is located upstream of the −35 box (DNA3), and the other is located around the −10 box (DNA4) [Fig. 3 ▸(*a*)]. To confirm the interaction of DNA with the *Mt*HigA3 antitoxin, we performed an EMSA. The DNA was resolved on a gel in the absence or in the presence of the protein. The DNA concentration was maintained at 0.01 µ*M*, and the protein concentration was increased from 0 to 0.6 µ*M*, resulting in 1:0, 1:10, 1:20, 1:30, 1:40, 1:50 and 1:60 molar ratios (DNA:protein). We observed a band upshift for DNA2 only, confirming the binding of DNA2 with *Mt*HigA3 [Fig. 3 ▸(*b*)]. The result indicates that the −10 box could act as the *higA3* operator, and *Mt*HigA3 binds specifically to this sequence.

We additionally conducted ITC experiments to quantify the interaction between *Mt*HigA3 and DNA2 (−10 box). The binding stoichiometry (*n*) was calculated as 0.910 ±0.004 using a one-site model. This indicates that one dimer of *Mt*HigA3 binds to one DNA2 fragment. The binding affinity (*K*_d_) was determined to be 11.3 ±1.8 n*M*, indicating high affinity. The interaction between DNA2 and *Mt*HigA3 is exothermic, with a negative change in enthalpy (Δ*H* = −8.83 ±0.09 kcal mol^−1^) and a favourable entropy (*T*Δ*S* = 2.03 kcal mol^−1^) [Fig. 3 ▸(*c*)].

### 3.4. Overall structure of *Mt*HigA3 bound to DNA   

**Figure 4:**
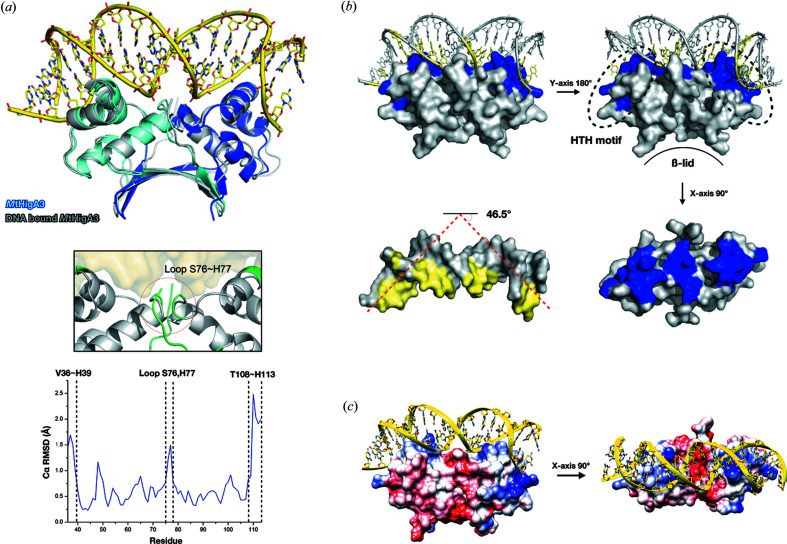
Overall structure of *Mt*HigA3 bound to DNA. (*a*) Superimposed cartoon representation of *Mt*HigA3 bound to DNA and the *Mt*HigA3 dimer structure (produced by matching the *Mt*HigA3 dimer of both models). The *Mt*HigA3 dimer is coloured cyan and blue. *Mt*HigA3 bound to DNA is coloured grey. The DNA fragment of *Mt*HigA3 bound to DNA is coloured yellow. Plot of Cα r.m.s.d. of the *Mt*HigA3 dimer and *Mt*HigA3 bound to DNA to compare Cα movement after DNA binding. N-terminal (Val36–His39), loop region (Ser76–His77) and C-terminal (Thr108–His113) residues show relatively large deviations. The region showing more than 1 Å r.m.s.d. is drawn in the black box and highlighted in green. (*b*) Cartoon and surface representation of *Mt*HigA3 bound to the DNA structure in diverse orientations. The regions of *Mt*HigA3 and DNA structure, which involve contact with each other, are coloured blue (*Mt*HigA3) and yellow (DNA). Bent DNA in *Mt*HigA3 bound to the DNA structure is marked as a red line. Promoter DNA is dramatically bent by the binding of *Mt*HigA3 (46.5°). (*c*) Superimposed *Mt*HigA3 dimer and DNA structure of *Mt*HigA3 bound to DNA. The electrostatic surface potential of the *Mt*HigA3 dimer is coloured between −10 kT e^−1^ (red) and 10 kT e^−1^ (blue) in two orientations rotated by 90°. The DNA structure of *Mt*HigA3 bound to DNA is presented as a cartoon representation. DNA binds to the positively charged region of the *Mt*HigA3 dimer.

Since DNA2 showed clear binding in the EMSA and ITC experiments, we tried to co-crystallize *Mt*HigA3 with DNA2. The dsDNA was incubated for 1 h at room temperature with dimeric *Mt*HigA3 before crystallization. *Mt*HigA3 bound to DNA was found to crystallize in space group *C*2 with the *Mt*HigA3 homodimer and 20 bp dsDNA in the asymmetric unit. Since the DNA duplex used for co-crystallization was pseudo-palindromic, it was not in a strict twofold symmetry. However, the homodimer and dsDNA dyad forms (dyad of the *Mt*HigA3 monomer and single-stranded DNA) correspond to crystallographic dyads. The same dataset was successfully reintegrated and determined in space group *C*2221 which lacks the crystallographic dyad. This is probably a consequence of the random bimodal orientation of the complex around the dyad (Becker *et al.*, 1998 ▸). Detailed DNA structural analysis was difficult, but we could identify the structures of *Mt*HigA3 residues and DNA nucleotides. The electron-density map of the N-terminal 35 amino acids of *Mt*HigA3 bound to DNA was missing, probably due to protease activity (Hadži *et al.*, 2017 ▸; Hadži *et al.*, 2013 ▸). The whole topology of *Mt*HigA3 bound to DNA is similar to that of the *Mt*HigA3 dimer. The overall structures of the *Mt*HigA3 dimer and *Mt*HigA3 bound to DNA are very similar (0.81 Å r.m.s.d. for 78 corresponding Cα atoms of chain A) [Fig. 4 ▸(*a*)]. However, the N-terminal residues of chain A of the *Mt*HigA3 homodimer (Asp34 and Ala35) show a relatively high r.m.s.d. (1.45–2.48 Å). These residues are missing in the structure of *Mt*HigA3 bound to DNA. Another region that shows high r.m.s.d. is the loop region (Ser76 and His77) which is compactly positioned in the DNA minor groove, located at the centre of the DNA [Fig. 4 ▸(*a*)].

The interactions between DNA and the *Mt*HigA3 dimer occur mainly through HTH motifs. Similar to other HTH motifs, the major groove of DNA directly interacts with HTH motifs of the *Mt*HigA3 dimer. The recognition α3-helix is inserted in the major groove, and the α2 and the loop that links the α2- and α3-helices support the contacts with the DNA backbone. In the structure of *Mt*HigA3 bound to DNA, the *Mt*HigA3 homodimer and DNA show an average interface area of 341.8 Å, which is relatively small. Since HTH motifs are positively charged, this motif binds to negatively charged DNA stably via an overall charge–charge interaction [Fig. 4 ▸(*c*)]. Fig. 4 ▸(*b*) shows the overall structure and conformation of DNA with the dimeric protein. The major groove of the DNA is largely responsible for binding and/or interacting with the HTH motifs of the dimeric protein [Fig. 4 ▸(*b*)]. The DNA-bound complex structure indicates that the N-terminal region does not mediate DNA contact. The data suggest that the N-terminal end (34 residues), although susceptible to protease activity, is not involved in DNA interaction.

Interestingly, unusual bending of DNA was observed in *Mt*HigA3 bound to DNA. The DNA fragment bends towards the minor groove with an overall bending angle of 46.5°, as calculated by the *CURVES* server (Lavery *et al.*, 2009 ▸) [Fig. 4 ▸(*b*)]. The average width of the minor groove in canonical B-DNA is 6 Å, but in the structure of *Mt*HigA3 bound to DNA, the width of the minor groove at the A-tract region (bases 9–12) located at the centre of the DNA is narrow (∼3.7 Å) (Fig. S4). The A-tract region in the DNA core induces the bending of DNA through the interaction between the proteins and the phosphodiester backbone in DNA, causing compression of the minor groove (Ball *et al.*, 2012 ▸; Hizver *et al.*, 2001 ▸). The minor-groove width at bases 16 to 18 (∼2.5 Å) is also compressed, which stablizes the structure of *Mt*HigA3 bound to DNA.

When the structure of *Mt*HigA3 bound to DNA was compared with that of the *Mt*HigA3 dimer, we found that the distance between the HTH motifs of each monomer was changed. The distance measured from Q64 of each monomer on the α3-helix was increased by 1.9 Å in the *Mt*HigA3 bound to DNA compared with the *Mt*HigA3 dimer (from 37.3 Å to 39.2 Å). This increase is due to a shift of the *Mt*HigA3 monomer in *Mt*HigA3 bound to DNA, which has been reported previously in *Pv*HigA (Schureck *et al.*, 2019 ▸). The results suggest that rearrangement of the dimer structure is required for DNA binding, providing a wider space between the HTH motifs of each monomer.

### 3.5. Characterization of the DNA-binding residue of *Mt*HigA3 by NMR titration   

**Figure 5:**
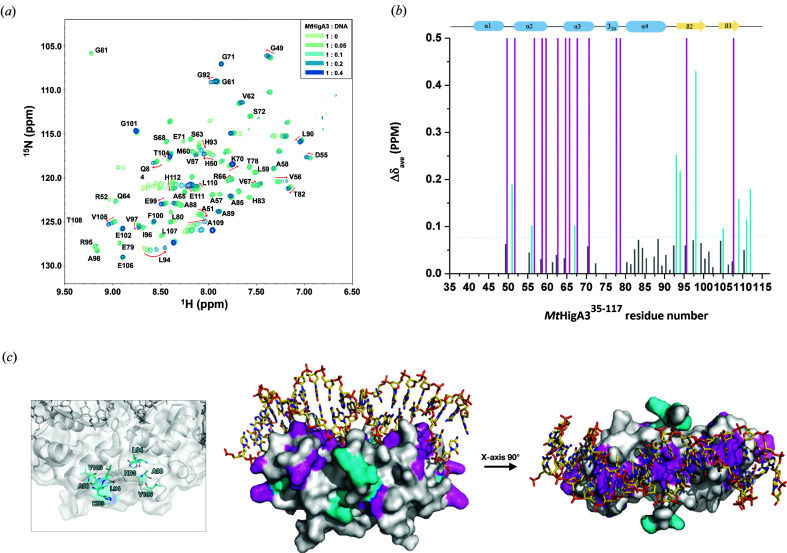
NMR titration of *Mt*HigA3^35–117^ and its promoter DNA. (*a*) Overlaid [^1^H-^15^N] TROSY–HSQC titration spectra of 0.4 m*M* ^15^N-labelled *Mt*HigA3^35–117^ with increasing concentrations of promoter DNA. (*b*) CSP analysis of *Mt*HigA3^35–117^ upon binding to the promoter DNA. CSPs > average CSP values (0.075) are coloured cyan, CSPs < average CSP values (0.075) are coloured grey, and the peaks that disappeared upon the addition of DNA are presented as magenta bars. Secondary structural elements of *Mt*HigA3^35–117^ are represented above the plot by blue cylinders (α-helices) and yellow arrows (β-strands). (*c*) The residues showing higher CSP values than the average CSP value in the β-lid (His93, Leu84, Ala98 and Val105) are shown as sticks and coloured cyan in the box. For a clear display, *Mt*HigA3 bound to DNA is presented as a cartoon diagram in surface view (left). Chemical shift mapping based on the CSP data from (*b*) on the crystal structure of *Mt*HigA3 bound to DNA in two orientations rotated by 90°. The DNA structure is represented using sticks for clarity.

The interaction between *Mt*HigA3 and DNA was revealed by the crystal structure of *Mt*HigA3 bound to DNA. To identify the binding properties of *Mt*HigA3 to DNA in the solution state, which is close to the natural state, we performed NMR titration experiments. Initially, we tried to assign the full sequence of *Mt*HigA3, but the NMR resonances of full-length *Mt*HigA3 were not well dispersed. This might be caused by the flexibility of the N-terminus of *Mt*HigA3. Based on our *Mt*HigA3 crystal structure, we eliminated 34 amino acids at the N-terminus of *Mt*HigA3 from the construct. N-terminus-truncated *Mt*HigA3, including the His_6_-tagged C-terminus (*Mt*HigA3^35–117^), shows a well dispersed 2D-[^1^H-^15^N] TROSY–HSQC spectrum. Backbone assignment of *Mt*HigA3^35–117^ (with the exception of the residues Ala35–Leu48, Gln53, Ala54, Lys69, Gly73, Leu75–His77, Tyr86, Asn103, Leu107 and His113–His117) could be performed and was used for the NMR titration experiment of 0.4 m*M* ^15^N-labelled *Mt*HigA3^35–117^ with promoter DNA [Fig. 5 ▸(*a*)]. By monitoring the HSQC spectra of *Mt*HigA3^35–117^ with increasing DNA concentration, line broadening and chemical shift changes were observed. The signal broadening of the NMR spectrum implies slow-to-intermediate exchange on the NMR time scale, a characteristic of moderate-to-high binding affinity. Most NMR signals upon the addition of DNA show weakening or disappearance. These signals correspond to residues that are in close contact with DNA in the crystal structure of *Mt*HigA3 bound to DNA [Figs. 5 ▸(*b*) and 5 ▸(*c*)]. The peaks of *Mt*HigA3^35–117^ which disappeared in the titration experiment, namely, the peaks for His50, Arg52, Ala57, Leu59, Met60, Ser63, Ala65, Arg66, Ser68, Glu71, Thr78, Glu79, Ile96 and Thr108, were located around the HTH motif consisting of the α2- and α3-helices (53–72), which interact with the major groove of the DNA. The residues Thr78 and Glu79, located at the loop between η1 and α4, interact with the minor groove of the A-tract region in the DNA. The residues His50 and Arg52 are located in the loop between the α1- and α2-helices, which is in front of the HTH motif. Their tight binding with both ends of the DNA could be related to the curvature of the whole DNA molecule.

The most highly perturbed peaks presenting higher CSP values than the average CSP value (0.075) were for Ala51, Val56, Val67, His93, Leu94, Ala98, Val105, Glu111 and His112. These residues are located in the α2- and α3-helices of the HTH motif and in the β2- and β3-strands that form the β-lid. Generally, peak shifting in the NMR spectrum can be attributed to the fast exchange phenomenon on the NMR time scale, which indicates low-to-moderate binding affinity in solution state. Although the β2- and β3-strands participating in the dimerization of *Mt*HigA3 are not directly involved in DNA binding in the crystal structure, the NMR titration result indicates that this region is associated with DNA interaction. This implies that the dimerization mode was affected by DNA binding.

## 4. Discussion   

Tuberculosis caused by *M. tuberculosis* is a prevalent disease worldwide, and unsuccessful treatment resulting from antibiotic resistance and tolerance of *M. tuberculosis* is a risk factor for global public health care. TA systems related to antibiotic resistance and tolerance are emerging as new antibiotic drug targets of TB. Among the six types of TA systems classified to date, *M. tuberculosis* has numerous type II TA systems. We studied the *Mt*HigA3 type II TA system protein, a cognate antitoxin of the *Mt*HigB3 ribonuclease toxin. *Mt*HigA3 is predicted to be a transcriptional regulator that autoregulates the *higBA3* operon by binding to the operator as an antitoxin itself or via complex formation with the toxin *Mt*HigB3, similar to other type II TA systems.

We firstly present the structure of HigA3 of *M. tuberculosis*, possessing unique characteristics compared with other HigA homologues, with the exception of the HTH motif (Figs. 1 ▸ and 2 ▸). In particular, the presence of a β-lid at the C-terminus of *Mt*HigA3 is an unusual structural feature of HigA homologues. From a structural perspective, *Ec*HipB, which has a small β-lid at the C-terminus, presents a rather similar structure to *Mt*HigA3. This structural difference in the same TA family may indicate structural diversity within the TA system. HigBA homologue complexes do not share similar complex interfaces. Briefly, the N-terminal region, being intrinsically disordered, is not observed in *Vc*HigA2 and in *Mt*HigA3 as a solitary antitoxin structure, whereas the structure of *Vc*HigA2 in the *Vc*HigBA2 complex has a long loop, one α-helix and one β-strand at the N-terminus forming a complex interface.

We have shown that the HTH motif mainly contributes to the DNA binding of *Mt*HigA3 by determining the complex structure of *Mt*HigA with operator DNA and by NMR titration experiments of *Mt*HigA3^35–117^ with DNA. The HTH motifs of dimeric proteins are responsible for binding to DNA and are associated with the characteristic bending of DNA. Residues with large chemical shift changes or residues that disappeared in the NMR titration, corresponding to the DNA-binding residues, are located at the HTH motif (Ala51, Arg52, Ser63, Ala65, Arg66 and Glu71). The structure of the *Mt*HigA3 dimer in the complex structure is similar to that of the *Mt*HigA3 dimer. However, the distance between each monomer is increased in the complex structure, and the angle of the *Mt*HigA3 dimer is changed.

**Figure 6:**
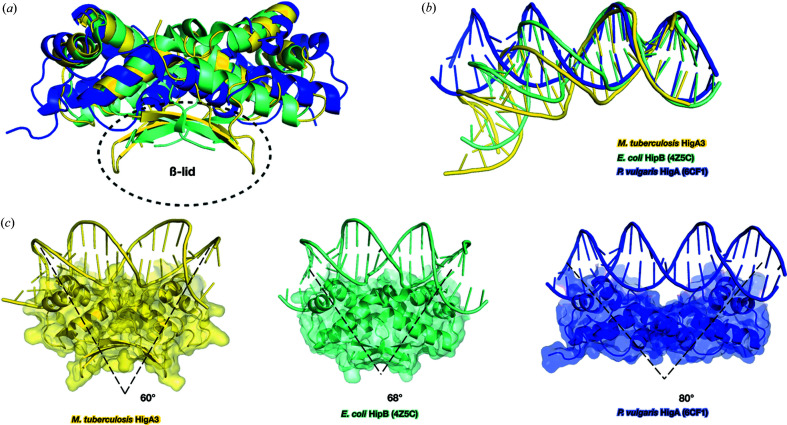
Structural comparison of *Mt*HigA3 bound to DNA and its homologues. (*a*) Cartoon representation of *Mt*HigA3 bound to DNA (yellow), *Pv*HigA bound to DNA (blue) and *Ec*HipB bound to DNA (cyan). The C-terminus of the antitoxin which differs markedly in comparison is marked with a black dotted circle. Only the protein structures in these DNA–protein complexes are shown for clarity. (*b*) The superimposed cartoon represents the DNA structure of *Mt*HigA3 bound to DNA (yellow), *Pv*HigA bound to DNA (blue) and *Ec*HipB bound to DNA (cyan). Only the DNA structures in these DNA–protein complexes are shown for clarity. (*c*) Structure of *Mt*HigA3 bound to DNA, *Pv*HigA bound to DNA and *Ec*HipB bound to DNA. The antitoxins are presented as cartoon diagrams in surface view. Dimer angles between central stalks are indicated.

By comparing the structure of *Mt*HigA3 bound to DNA with those of *Pv*HigA bound to DNA and *Ec*HipB bound to DNA, we found that the quaternary structure of the antitoxin affects the curve of DNA bending. When the structures of *Mt*HigA3 bound to DNA, *Pv*HigA bound to DNA and *Ec*HipB bound to DNA were aligned based on the antitoxin monomer, the curvature of the DNA increased as the dimer angles decreased [Fig. 6 ▸(*b*)]. The major structural difference between these DNA–protein complexes was located at the C-terminus of the antitoxins, which take part in protein dimerization. *Mt*HigA3, *Pv*HigA and *Ec*HipB show different tertiary structures at the C-terminus, located on the opposite side of the DNA-binding interface. *Pv*HigA lacks a β-strand at the C-terminus and is dimerized only through a hydrophobic core composed of α-helices. While *Ec*HipB has two β-stranded β-lids at the C-terminus, *Mt*HigA3 shows a broad β-lid formed by four β-strands at the C-terminus and one β-strand at the N-terminus [Figs. 6 ▸(*a*) and 6 ▸(*c*)]. The difference in the three structures revealed that the higher the amount of secondary structure at the C-terminus, the tighter the binding network of the antitoxin dimer. This difference induces a change in the quaternary structure of antitoxin dimers by regulating the dimer angle of the antitoxin structure. This change in the quaternary structure of the antitoxin could induce the bending of DNA.

In this study, we obtained the first structural data for *M. tuberculosis* HigA3. From this result, we found that, even though HigA homologues have structurally similar HTH motifs, the overall structures are different from each other. We also obtained the crystal structure of *Mt*HigA3 bound to DNA and information for the binding of *Mt*HigA3 with DNA by NMR titration experiments. From EMSA experiments, we confirmed that *Mt*HigA3 recognizes DNA sequences specifically. Our research could provide structural information for understanding *higBA3* operon regulation. Based on these structural data, the development of peptides mimicking and/or inhibiting the *Mt*HigA3–DNA binding interface may be possible. These results may provide useful information for structure-based antibiotic development.

## Supplementary Material

Supporting information file. DOI: 10.1107/S2052252520006466/lz5038sup1.pdf

PDB reference: MtHigA3, 6ltz

PDB reference: DNA-bound MtHigA3, 6lty
