# Supplementary material for: Induced DNA bending by unique dimerization of HigA antitoxin
Source: IUCrJ. 2020 Jun 26;7(Pt 4):748–60. doi: 10.1107/S2052252520006466 (PMC7340258; doi:10.1107/S2052252520006466)
Supplement: Supplementary file 1 [file m-07-00748-sup1.pdf]

IUCrJ

Volume 7 (2020)

Supporting information for article:

Induced DNA bending by unique dimerization of HigA antitoxin

Jin-Young Park, Hyo Jung Kim, Chinar Pathak, Hye-Jin Yoon, Do-Hee Kim, Sung Jean Park and Bong-Jin Lee

Table S1 Oligonucleotide primers.

Oligo	Sequence
*Mt*HigA3 forward	GGAATTCCATATGACCATGGCCCGCAACTGGCG
*Mt*HigA3 reverse	CCGCCGCTCGAGGGCGGTCAGCTCGACAGTATTT
*Mt*HigA3$^{35\text{-}117}$ forward	GGAATTCCATATGGCCGTCCTGGCGCACCG
*Mt*HigA3$^{35\text{-}117}$ reverse	CCGCCGCTCGAGGGCGGTCAGCTCGACAGTATTT

Table S2 Data collection and refinement statistics.

	*Mt*HigA3	DNA bound *Mt*HigA3
Data collection		
Diffraction source	BL-5C, PLS	BL-5C, PLS
Wavelength (Å)	0.9794	0.9796
Detector	ADSC quantum 315*R* CCD	ADSC quantum 315*R* CCD
Space group	*I*4	C2
a, *b*, *c* (Å)	84.23, 84.23, 61.43	74.61, 101.81, 58.03
α, β, γ (°)	90, 90, 90	90, 90.05, 90
Resolution range (Å)	50–1.97 (2.0)	50-3.27 (3.36)
R_{merge} (%)[a]	5.6 (17.8)	6.2 (55.9)
Completeness (%)	100 (100)	99.6 (98.4)
Redundancy	6.7 (6.3)	2.6 (2.5)
$\langle I/\sigma(I) \rangle$	41.5 (9.095)	27.3 (2.975)
$CC_{1/2}$	0.99275 (0.978)	1 (0.886)
Refinement		
No. of reflections	15192	6640
Final R_{cryst}	0.189	0.277
Final R_{free}	0.234	0.326
No. of non-H atoms		
Protein/ligand	1141/41	1168/0

DNA	0	820
Water	81	0
R.m.s. deviations		
Bonds (Å)	0.004	0.008
Angles (°)	0.803	1.340
Average *B* factors (Å^2)		
Protein/ligand atoms	40.28/53.21	130.3
DNA	0	182.7
Water	45.18	0
Wilson B factor	30.67	135.8
Ramachandran plot		
Most favoured (%)	99.33	74.34
Allowed (%)	0.67	21.05
Disallowed region (%)	0	4.61
PDB accession code	6LTZ	6LTY

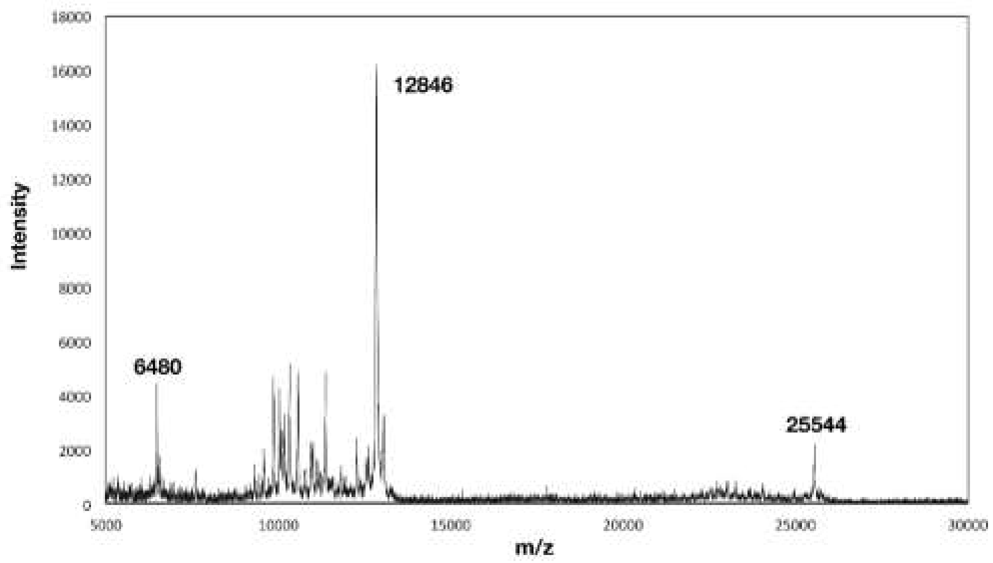

Figure S1 MALDI-TOF mass spectrum of purified *Mt*HigA3. A major peak was observed at 12846 Da in the spectrum, corresponding to the calculated mass of full-length *Mt*HigA3.

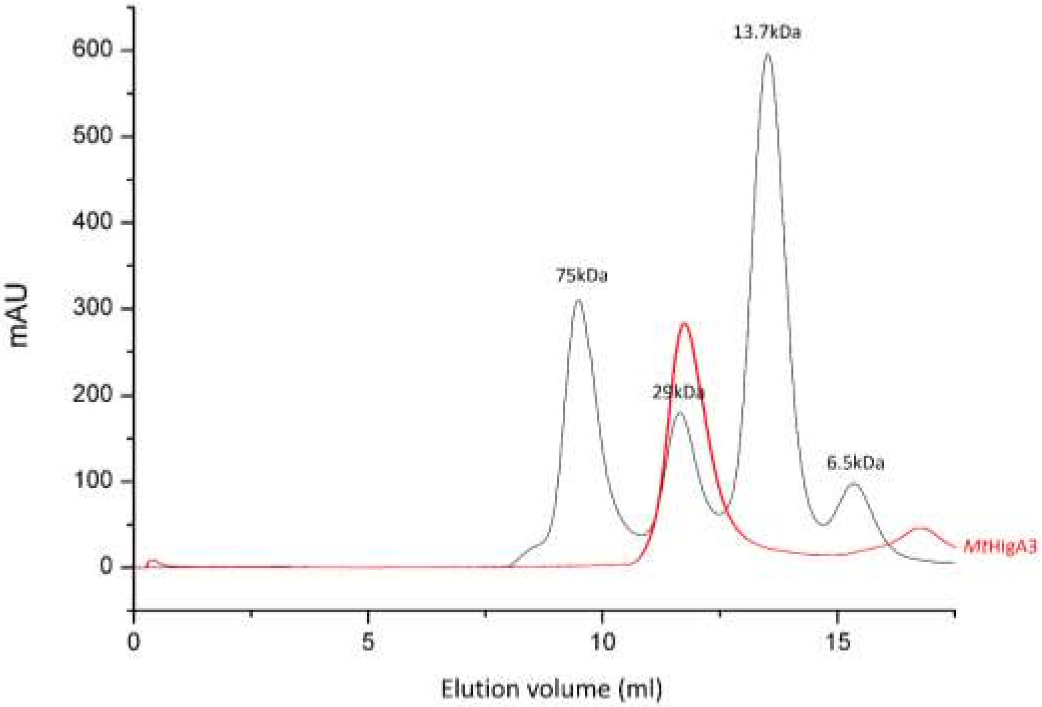

Figure S2 *Mt*HigA3 forms a dimer in the solution state. Size-exclusion chromatography (SEC) column profile for *Mt*HigA3. The protein eluted as one peak corresponding to the dimeric state of the *Mt*HigA3 protein (shown as a red colored trace). The standard low-molecular-weight calibration kit is shown in grey for comparison.

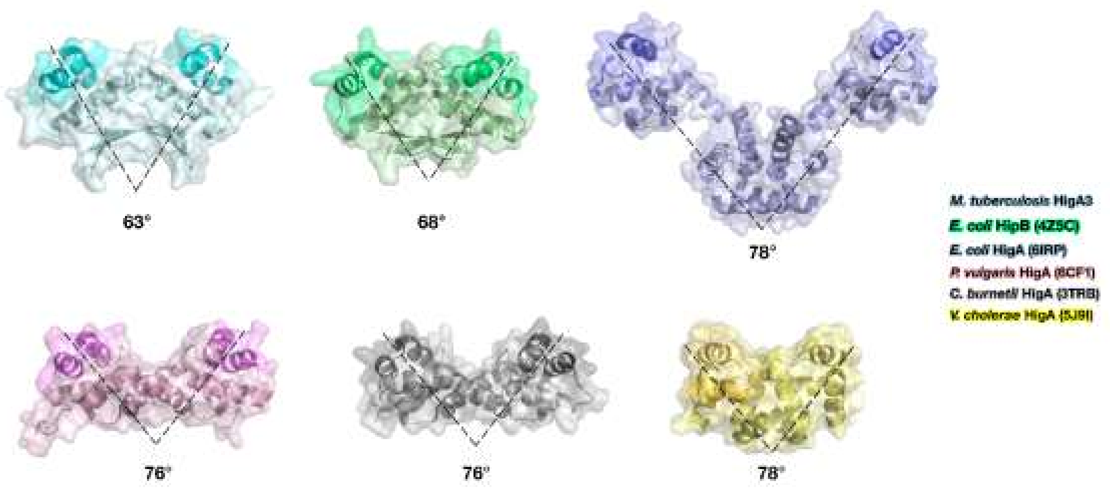

Figure S3 Structure of *E. coli* HipB (lime green, PDB code: 4Z5C), *E. coli* HigA (slate, PDB code: 6IRP), *P. vulgaris* HigA (pink, PDB code: 6CF1), *C. burnetii* HigA (grey, PDB code: 3TRB) and *V. cholerae* HigA (yellow, PDB code: 5J9I). The adjacent chain of each dimer is colored lighter, and HTH motifs are colored darker. The antitoxin dimers are presented as cartoon diagrams in surface view. Dimer angles between central stalks are indicated.

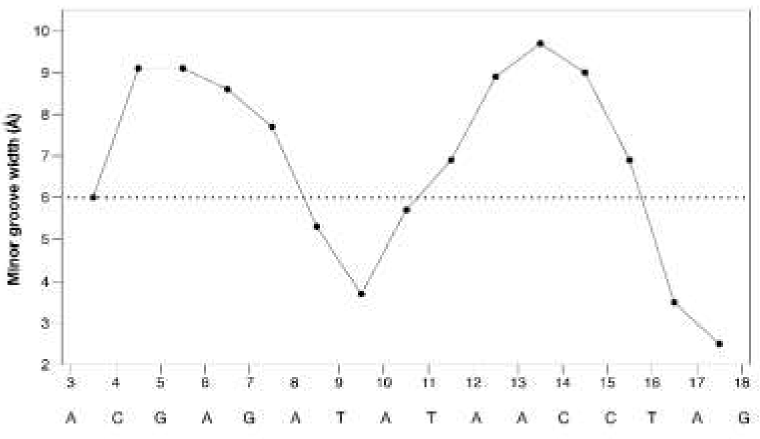

Figure S4 DNA distortion in *Mt*HigA3 bound to DNA. Minor groove widths are plotted over the length of the DNA. Groove parameters were analysed using the CURVES server. Dashed lines indicate canonical groove widths for B-DNA.
